# Malnutrition among hospitalized children 12–59 months of age in Abyan and Lahj Governorates / Yemen

**DOI:** 10.1186/s40795-022-00574-z

**Published:** 2022-08-12

**Authors:** Ali Ahmed Al-Waleedi, Abdulla Salem Bin-Ghouth

**Affiliations:** 1 Department of Public Health and Community Medicine, Faculty of Medicine, University of Aden, Aden, Yemen; 2 Department of Community Medicine, Hadharamout University College of Medicine (HUCOM), Hadhramout University, 8892 Mukalla, Fwah Yemen

**Keywords:** Malnutrition, Sick children, Stunting, Wasting

## Abstract

**Background:**

The analysis of acute malnutrition in 2018 for the Integrated Phase Classification of Food Security in Yemen shows that high malnutrition rates are present in Abyan governorate (23%) and Lahj governorate (21%). This analysis was community based addressed all children and mostly due to problems related to food intake. The role of diseases was not yet addressed in Yemen. The aim of this study is to assess acute and chronic malnutrition among hospitalized children at 12–59 months of age in Lahj and Abyan governorates in Yemen.

**Methodology:**

A cross-sectional, multi-center study is designed. The assessment of the nutritional status was measured by standardized anthropometry of 951 sick children at 12–59 months of age.

**Results:**

The prevalence of Global acute malnutrition (GAM) among the sick children seeking care in health facilities in Lahj and Abyan is 21%. More specifically; the prevalence of moderate acute malnutrition (MAM) is 15.1% while the prevalence of severe acute malnutrition (SAM) is 6.2%. The prevalence of acute malnutrition (wasting) among the studied sick children in lahj is 23.4% while in Abyan is 19.3%. The prevalence of MAM in Lahj is 17.7% and the prevalence of SAM is 5.7%. The prevalence of acute malnutrition (wasting) in Abyan is 12.6% while the prevalence of SAM in Abyan is 6.7%. The prevalence of acute malnutrition among male children (25.2%) is significantly higher than among female children (17.5%). The prevalence of the chronic malnutrition (Stunting) in the studied sick children is 41.3%; the prevalence of stunting in Lahj is 41% while in Abyan is 41.7%.

**Conclusions:**

High acute and chronic malnutrition rates were identified among sick children seeking care in health facilities in lahj and Abyan, and higher than the SPHERE indicators of malnutrition. Boys are more exposed than girls to acute and chronic malnutrition.

## Introduction

Malnutrition in children is of high concern in developing countries like Yemen. However, malnutrition is multifactorial. Malnutrition in low-income countries is often, but not solely, be attributable to limited access to food and/or medical care, it is often triggered by disease [1]. In one study among 3101 hospitalized children in nine countries in sub-Saharan Africa in 2019; it was found that 24.6% of the hospitalized children had moderate wasting, and 39·3% had severe wasting with death rate of 11·3% [2].

Most of the local and international reports described the situation in Yemen is the worst humanitarian crises in the world. Malnutrition among Yemeni children is one of the painful crises. In 2015, UNICEF's report concludes that a striking ten of Yemen's 22 governorates are on the edge of famine, as defined by the five-point Integrated Food Security Phase Classification (IPC) scale [3]. In 2017; a study published in the lancet indicated that according to organizations working to end hunger, about 370 000 of Yemen's children are suffering from severe malnutrition. Additionally, one million children younger than five years old are at risk of acute malnutrition [4].

The rate of child malnutrition in Yemen is one of the highest in the world and the nutrition situation continues to deteriorate. World food program (WFP) reported that about one third of families have gaps in their diets, and hardly ever consume foods like pulses, vegetables, fruit, dairy products or meat. Malnutrition rates among children in Yemen remain among the highest in the world, with 2.3 million children under five years requiring treatment for acute malnutrition [5].

Another study among children under five years of age identified that the high malnutrition level (the prevalence of stunting was 47%, wasting was 16%, and underweight was 39%) [6].

Recent study in 2022 reported that more than 2.3 million children under the age of five in Yemen suffer from acute malnutrition. Approximately 450,000 are expected to suffer from severe acute malnutrition and may die if they do not receive urgent treatment [7].

Cases of acute malnutrition among children under five have risen to the highest levels recorded in parts of Yemen. More than half a million cases recorded in the southern districts. Analysis of acute malnutrition in 2018 in Yemen for the Integrated Phase Classification of Food Security issued by the Organization Food and Agriculture of the United Nations (FAO), the United Nations Children's Fund (UNICEF), the World Food Program and their partner identified high rate of malnutrition. The most affected areas included in this analysis are Abyan governorate (23%), Lahj governorate (21%) [8], in another two studies the global acute malnutrition in Abyan was 10% [9]. And in lahj was 27.3% [10].

Malnourished children are more vulnerable to illnesses, including diarrhea, respiratory infections, and malaria, which are a major concern in Yemen [11] Disease-related malnutrition in children is the consequence of different factors. For example, food intake due to anorexia, feeding difficulties or the effects of medications or due to the hyper metabolic state caused by the underlying disease [12–14].

Identification of malnutrition among hospitalized children is important because most pediatricians have no concern on the impact of malnutrition on the clinical outcome of the sick child. Mostly, they neglect the malnutrition as a determinant of the disease prognosis. This study aimed is to assess the malnutrition among sick children in two governorates in the southern Yemen of high malnutrition prevalence. The specific objective is to assess the prevalence of acute and chronic malnutrition among children aged 12 to 59 months seeking outpatient care and their association with governorate, health facility, gender, residency, family income, availability to drinking water.

### Methodology

A cross-sectional, multi-center study was designed in to determine the prevalence of malnutrition and related morbidity among hospitalized children 12–59 months of age in Abyan and Lahj governorates.

**Fig. 1:**
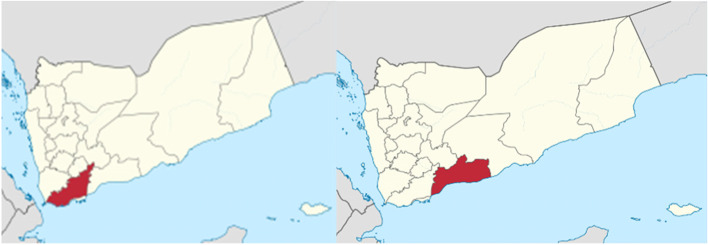
Map of Yemen the coloured areas are Lahj (left) and Abyan (Right) governorates. Source: https://en.wikipedia.org/wiki/Lahij_Governorate & https://en.wikipedia.org/wiki/Abyan_Governorate

In Yemen, there are 22 governorates; since The Civil War in 2015; twelve governorates in the south are under the control of International recognized government (IRG) including lahj and Abyan governorates. The total population of Yemen in 2021 is 31,153,000 people. The total population of Lahj in 2021 is 1,070,000 people including 115,685 children at 12–59 months while population of Abyan is 609,000 people including 55,547 children at age of 12–59 months [15]. In each governorate there is one government hospital and, in each district, there is one district hospitals. In each district there is a main city and a group of villages, in a big village there is one primary health care (PHC) center. Figure 1

The assessment of the nutritional status was measured by standardized anthropometry at attendance in outpatient clinic. Wasting measured by Weight for height/length or MUAC and stunting measured by Hight/length for age (SDS, WHO reference) are the primary outcome variables. Gender, Family residency, family income, availability of drinking water in the house are the independent variables.

The study population are children at 12–59 months of age who attend the health facility to seek care for certain health problem. Mothers were interviewed while a trained nurse measured the weight and height and mid upper arm circumference (MUAC) of the sick child.

From each governorate; five health facilities were selected. These facilities were: the main governorate hospital, two district hospitals and two health centers from two different villages in two different districts. There is only one governorate hospital in every governorate. The other four health facilities were selected based on the selection of the districts. From every governorate; two districts were selected by simple random sampling out of 12 districts in every governorate. From each district we select the district hospital (it is only one district hospital in each district) and two villages out of 8–12 villages in each district. From each village we select the village PHC center (it is one center in every village).

Data was collected through a group of enumerators and two field supervisors. Training of two days were conducted in Lahj (Al-Hottah city) in 28^th^ of February, 2022 and in Abyan (Zunjibar city) in 3^rd^ of March, 2022 where enumerators were trained about the questionnaire and the selection of the targeted children (sick children seeking care in the selected health facility within 12–59 months of age). IT personal trained the enumerators about applying the KOBO toolbox and upload the digital questionnaire to their mobiles. This method is most effective method to make the research team monitors in daily basis the process of data collection.

### Sample size calculation

The formula that is used to calculate the sample size is Danieal formula of cross-sectional study in infinite population [16].

The following simple formula (Daniel, 1999) can be used:

$$N=\frac{{z}^{2}P\left(1-p\right)}{{d}^{2}}$$

where $N$ = sample size, $z$ = z statistic for a level of confidence (1.96), $P$= expected prevalence or proportion, here is 10% based on the prevalence of malnutrition in Abyan[9], and $d$ = precision ($d$ = 2). Accordingly; the sample size will be:

$$N=\frac{1.962*0.10*\left(1-0.10\right)}{{2}^{2}}=3457/4=864$$

We add 10% to avoid non response, so the final sample size was 864 + 86 = 950.

The samples size was distributed equally for Lahj and Abyan (475 from each) then was distributed proportional to health facility category and based on the follow of the sick children to health facilities (37% in hospitals, 21% in district hospitals and 42% in health centers). So; the sample was distributed as 175 children from each governorate hospital, 100 children from each district hospital and 50 children from each health center.)

### Anthropometric measurements [17]

- Weight: Children were weighed standing on the weight scale to the nearest 0.1 kg. For the children who could not stand, weight was measured in infant weight scale.
- Height/Length: Height and length of children were measured using height scale and recorded to the nearest 0.1 cm. Children equal or less than 87.0 cm were measured lying down, and children greater than or equal to 87.0 cm were measured in standing position.
- MUAC: Mid-upper arm circumference measurements were made using a flexible and non-stretch tape. MUAC measurements was taken on the mid-point of the left upper arm. All the selected sick children in the aged 12–59 months were measured to the nearest 0.1 cm. The MUAC is interpreted as both for graduated and color labeled. Red color [MUAC > 115 mm], and < 125 mm] were considered a moderately malnourished. While the green color [MUAC ≥ 125 mm] were categorized as normal according to WHO classification.

### Operational definition of the Outcome indicators [18]

Wasting: Weight‐for‐height (wasting) provides the clearest picture of acute malnutrition.

Moderate Acute Malnutrition (MAM) is identified by moderate wasting WFH ≤ -2 z score and ≥ -3 z‐score for children 0‐59 months (or for children 6‐59 months, MUAC < 115 mm and ≥ 125 mm). Table 1.

**Table 1:** Anthropometric measurements and indicators

Measurement	Indicator	Nutritional status
Weight-for-height index (W/H)	≥ -2 z-score	Normal nutrition status
	(< -3 z-score and/or oedema and/or < 115 mm (MUAC)	Acute severe malnutrition (SAM)
	WHZ ≥ -3 and < -2	Acute moderate malnutrition (MAM)
MUAC	> or = 125	Normal nutrition status
	< 125 and > or = 115	Acute moderate malnutrition (MAM)
	< 115	Acute severe malnutrition (SAM)
Stunting (Height for Age -HAZ)	≥ -2 z-score	Normal nutrition status
	3 z-score ≤ H/A < -2 z-score	Stunting (moderate)
	< -3 z-score	Stunting (Severe)

Severe Acute Malnutrition (SAM) is identified by severe wasting < -3 z‐score for children 12‐59 months (or for children 12‐59 months, MUAC < 115 mm) or the presence of bilateral pitting edema.

Global Acute Malnutrition (GAM) is the presence of both MAM and SAM in a population. A GAM value of more than 10 percent indicates an emergency. If GAM is exceeding 15% it is considered critical while at 11–14% is severe GAM and if GAM at level of > 5% and less than 10% is considered poor indicator.

Chronic malnutrition (Stunting) (Height-for-age Z score (HAZ)) The HAZ measure indicates if a child of a given age is chronically malnourished (stunted). The height-for-age index of a child from the studied population is expressed in Z-score (HAZ).

## The indicators

1. proportion of wasting (MAM, SAM and GAM) among the hospitalized children in Lahj and Abyan
   = Number of hospitalized children have MAM/ all hospitalized children under study.
   = Number of hospitalized children have SAM/ all hospitalized children under study.
2. Proportion of stunting (Chronic malnutrition) among the hospitalized children in Lahj and Abyan

 = Number of hospitalized children have stunting/ all hospitalized children under study.

### Statistical analysis

Data were transforming from the KOBO application data set to excel file, then to the statistical package for social sciences (SPSS) version 24. Descriptive statistics uses are mean, standard deviations, to describe the quantitative variables while frequency and percentages to describe the qualitative variables. Chi square test is used as a tool of inferential statistics to assess the significance of association between malnutrition and the independent variables of gender, residency, family income, availability of drinking water in the house. A cut of point of 0.05 was used for significant level. Fischer exact test is used alternatively to chi square test if the expected dells are less than 5.

For purpose of bivariate and logistic regression; the dependent variables were ree-classified into two categories: the acute malnutrition is reclassified into acute malnutrition and normal, where acute malnutrition included MAM and SAM children. The chronic malnutrition (Stunting) variable is a dependent variable is re-classified int o two categories: Chronic malnutrition (Stunting) and normal, where chronic malnutrition included moderate stunting and sever stunting. the socioeconomic variables of more than two categories are reclassified. The independent variables are re-classified in to two categories: For example, residency is re-classified to be (Resident and non-resident. The non-resident group includes IDPs, refugees and marginalized people. The availability of water of in the house is re-classified to available or not available. The availability of water includes the available and regular supply and availability with irregular supply. The outcome variables; acute and chronic malnutrition, each variable is classified into two categories. In all bivariate and logistic regression, the significant level is 0.05, Odds Ratio (OR) and 95% confidence interval (95%CI) are used to assess the strength of the association.

## Results

### Socio-demographic characteristics of the studied children

The total number of sick children seeking care in the selected 10 health facilities in Lahj and Abyan during the study period (1–13, March 2022) are 951 children at the age of 12–59 months. The mean age is 29.5 years old (± 14 years). The range from 12 to 59 months. A total of 491 female children (51.6%) while male children are 460 children (48.4%). There are 474 children form Lahj governorate (49.8%) and 477 children from Abyan governorate (50.2%). Most of the children's mothers are either illiterate (37.4%) or has primary /essential education (36/1%) while most of the fathers had primary or essential education (39.2%). About 64% of children's fathers are employed, but about 35% is unemployed; most of the mothers reported that their family monthly income is not enough (88.1%). About 75% of the children are of resident families and 23.7% of internal displaced people (IDPs). Most of the households of the children (62%) has irregular drinking water supply  Table 2.

**Table 2:** Socio-demographic characteristics of the sick children involved in the study (*N* = 951)

Socio-demographic characteristics		Number of the studied sick children (*N* = 951)	%
Sex	Male	460	48.4%
	Female	491	51.6%
Governorate	Lahj	474	49.8%
	Abyan	477	50.2%
Mother educational level	Illiterate	356	37.4%
	Primary/elementary/preparatory	343	36.1%
	Secondary/diploma	186	19.6%
	University +	66	6.9%
Father educational level	Illiterate	161	16.9%
	Primary/elementary/preparatory	373	39.2%
	Secondary/diploma	283	29.8%
	University +	134	14.1%
Father job	Unemployed	332	34.9%
	Employed	617	64.9%
	Student	2	0.2%
Family monthly income	Enough	112	11.9%
	Not enough	838	88.1%
Residency	resident	713	75%
	IDPs	225	23.7%
	Refugees	8	0.8%
	Muhamsheen	5	0.5%
Drinking water supply in the house	Available and Regular	262	27.5%
	Available but not regular	647	68%
	Not available	42	4.5%

### Prevalence of acute malnutrition among sick children

**Fig. 2:**
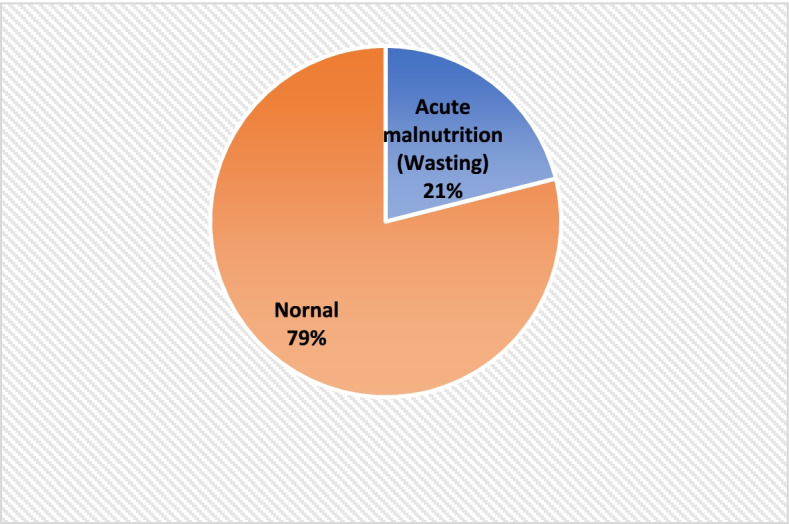
Prevalence of acute malnutrion (Wasting) among 951 sick children seeking care in health facilities in Lahj and Abyan, March 2022

**Fig. 3:**
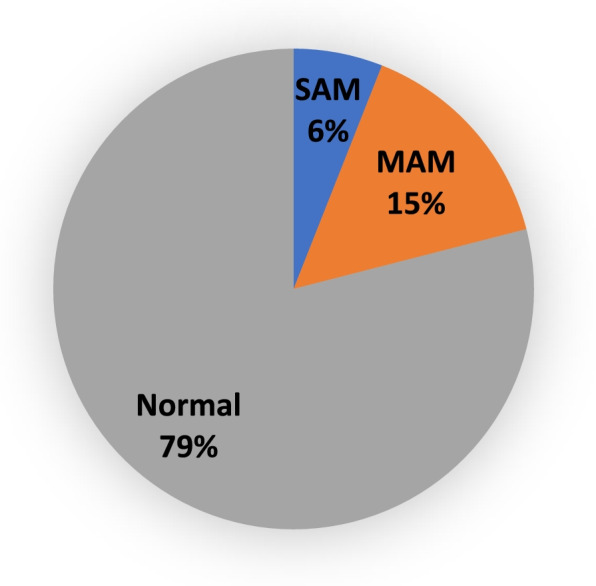
Prevalence of MAM and SAM among sick children seeking care in health facilities in Lahj and Abyan, March 2022

The prevalence of Global acute malnutrition (GAM) among the sick children seeking care in health facilities in Lahj and Abyan is 21% (203/951) Fig. 2. More specifically; the prevalence of moderate acute malnutrition (MAM is 15.1% (144/951) while the prevalence of severe acute malnutrition (SAM) is 6.2%. (59/951) Fig. 3

The prevalence of global acute malnutrition (wasting) among the studied sick children in lahj is 23.4% while in Abyan is 19.3%. The prevalence of MAM in Lahj is 17.7% and the prevalence of SAM is 5.7%. The prevalence of MAM among the studied sick children in Abyan is 12.6% while the prevalence of SAM in Abyan is 6.7% but these differences are not significant (*P*-value 0.113).

### The prevalence of the chronic malnutrition (Stunting)

The prevalence of the chronic malnutrition (Stunting) in the studied sick children is 41.3%; the prevalence of stunting in Lahj is 41% while in Abyan is 41.7%. The prevalence of moderate stunting in all the studied sick children is 24.3% and the prevalence of severe stunting is 17.2%. Prevalence of moderate stunting is higher in Abyan 26.4% while the prevalence of severe stunting is higher in Lahj (19.2%) but these differences are not significant (*P*-vale 0.117). Table 3. A 65 out of 951 children was found have concurrent form of malnutrition (wasting and stunting), giving the prevalence of concurrent forms of malnutrition to 6.8%

**Table 3:** Prevalence of acute malnutrition among the sick children seeking care in health facilities by governorate, March 2022

**Governorate**	**Category of Acute malnutrition**						**χ2**	*P*-value
	**MAM**	**%**	**SAM**	**%**	**GAM**	**%**		
Lahj (*n* = 474)	84	17.7%	**27**	5.7%	111	23.4%	0.24	0.113
Abyan (*n* = 477)	60	12.6%	**32**	6.7%	**92**	19.3%		
	**Category of chronic malnutrition (stunting)**						**χ2**	P-value
**Governorate**	Moderate stunting	**%**	Severe stunting	**%**	Overall stunting	**%**		
Lahj (*n* = 474)	103	21.7%	91	19.2%	194	41%	0.42	0.117
Abyan (*n* = 477)	126	26.4%	73	15.3%	199	41.7%		

### Variations in prevalence of malnutrition by gender

The prevalence of acute malnutrition among male children (25.2%) is significantly higher than prevalence of acute malnutrition among female children (17.5%). Moreover, Prevalence of MAM and SAM among males (17.6% & 7.6% respectively) are significantly higher than females (12.8% & 4.9% respectively) (P-value 0.004). The prevalence of stunting in males (45.3%) is significantly higher than females (37.7%). Moreover, Prevalence of moderate stunting and severe stunting among males (25.5% & 19.8% respectively) are significantly higher than females (22.6% & 15.1% respectively) (*P*-value 0.05). Table 4.

**Table 4:** Gender and malnutrition

**Sex**	**Category of Acute malnutrition**						**χ2**	*P*-value
	**MAM**	**%**	**SAM**	**%**	**GAM**	**%**		
**Male (***n* = 460)	81	17.6%	35	7.6%	116	25.2%	8.42	0.004*
**Female (***n* = 491)	63	12.8%	24	4.9%	86	17.5%		
	**Category of chronic malnutrition (stunting)**						**χ2**	*P*-value
**Sex**	Moderate stunting	**%**	Severe stunting	**%**	Overall stunting	**%**		
**Male (***n* = 460)	118	25.5%	90	19.8%	208	45.3%	5.99	0.005^*^
**Female (***n* = 491)	111	22.6%	74	15.1%	185	37.7%		

^*^Statistically significant at 0.05 significant level

### The socio-economic characteristics and malnutrition

For purpose of bivariate analysis; the socioeconomic variables are re-classified to be dichotomous variables for example; Residency and availability of water (see the statistical analysis section). In Table 5, the bivariate analysis is presented between the socio-economic factors (independent variables) and acute malnutrition (dependent variable). In bivariate analysis; only sex and type of health facility have significant association with acute malnutrition. Table 5 shows that high prevalence of acute malnutrition among the males (25.2%) is significantly higher than females (17.5%) (P-value 0.004). The prevalence of acute malnutrition in sick children seeking care in hospital clinics (24.8%) is significantly higher than prevalence of acute malnutrition in sick children seeking care in PHC centers (16.3%) (p-value 0.002). In Table 6, the bivariate analysis is presented between the socio-economic factors (independent variables) and chronic malnutrition (dependent variable). In bivariate analysis, sex, type of health facility and residency are significantly associated with chronic malnutrition (Stunting). Table 6 shows that the prevalence of stunting is higher in males (45.2%) than females (37.7%) (p-value 0.021). The prevalence of stunting in sick children seeking care in hospital clinics (47.8%) is significantly higher than prevalence of stunting in sick children seeking care in PHC centers (32.3%) (p-value 0.000). The prevalence of stunting in non-residents (IDPS, Refuges and marginalized groups) is significant higher (51.7%) than the prevalence of stunting in residents (37.9%) (P-value 0.000). Table 6.

**Table 5:** Bivariate analysis of association of acute malnutrition and socio-economic characteristics

Item		Acute malnutrition	Well nourished	Total	Prevalence of acute malnutrition	X^2^	*P*-value
Governorate	Lahj Abyan	111 91	363 386	474 477	23.4% 19.1%	0.27	0.113†
Sex	Male Female	116 86	344 405	460 491	25.2% 17.5%	8.42	0.004†*
Type of the Health facility	Hospital PHC centers	137 65	415 334	552 399	24.8% 16.3%	10	0.002†*
Residency	Resident	150	563	713	21%	0.07^*^	0.784
	Non-resident	52	186	238	21.8%		
Monthly family income	Enough	26	87	113	23%	0.24	0.625
	Not enough	176	662	838	21%		
Availability of drinking water in the house	available	193	716	909	21.2%	1^*^	0.551
	Not available	9	33	42	21.4%		

^†^Fischer exact test

^*^Significant, *P*-value < 0.05

**Table 6:** Bivariate analysis of association of Chronic malnutrition and socio-economic characteristics

Item		Stunting	Normal	Total	Prevalence of Stunting	X^2^	*P*-value
Governorate	Lahj Abyan	194 199	280 278	474 477	40.9% 41.7%	0.06	0.834
Sex	Male Female	208 185	252 306	460 491	45.2% 37.7%	5.56	0.021†*
Type of the Health facility	Hospital PHC centers	264 129	288 270	552 399	47.8% 32.3%	22.3	0.000*
Residency	Resident	270	443	713	37.9%	14	0.000†*
	Non-resident	123	115	238	51.7%		
Monthly family income	Enough	38	75	113	33.6%	3.13	0.084
	Not enough	355	483	838	42.4%		
Availability of drinking water in the house	available	370	539	909	40.7%	3.27	0.079†
	Not available	23	19	42	54.8%		

^†^Fischer exact test

^*^Significant, *P*-value < 0.05

In logistic regression, gender (male) and type of health facility (Hospital) are significantly associated with acute malnutrition (wasting). The odds of a male child have acute malnutrition is 60% higher than that of the female child (adjusted OR 1.6, 95% CI (0.161–0.797), *p* = 0.003) when other variables are held constant. Also, the odds of a child admitted into hospital having acute malnutrition is 0.45 lower than the child from the PHC facility child (adjusted OR 0.55, 95% CI (-0952–0.239), *p* = 0.001) when other variables are held constant. Regarding chronic malnutrition (Stunting); gender, type of health facility (Hospital) and residency are significantly associated with chronic malnutrition (stunting). The odds of a male child have chronic malnutrition is 40% higher than that of the female child (adjusted OR 1.4, 95% CI (0.082–0.625) when other variables are held constant, P-value 0.010. The odds of a child admitted into hospital having chronic malnutrition is 80% more than the child from the PHC facility (adjusted OR 1.8, 95% CI (0.345–0.947), *P*-value 0.000 when other variables are held constant. The odds of child of non-resident family having acute malnutrition are 39% more than the child from resident family (adjusted OR 1.39, 955 CI 0.009- 0.652. P-value 0.040) when other variables are held constant. Table 7

**Table 7:** Association of acute/chronic malnutrition and socio-economic characteristics as a result of logistic regression

Association of acute malnutrition (Wasting) and socio-economic characteristics					
			95%CI		
**socio-economic characteristics**	B	Exp(B)	Lower limit	Upper limit	*P*-value
Governorate (Lah)	0.328	0.720	-0.687	0.002	0.054
Sex (Male)	0.470	1.600	0.161	0.797	0.003*
Type pf health facility (Hospital)	0.589	0.555	-0.952	0.239	0.001*
Monthly family income	0.198	0.821	-0.677	0.343	0.444
Residency (Non -resident)	0.109	0.897	-0.510	0.290	0.581
Availability of drinking water	0.284	1.328	-0.600	1.095	0.446
**Association of chronic malnutrition (Stunting) and socio-economic characteristics**					
Governorate (Lahj)	0.080	1.084	-0.196	0.380	0.565
Sex (Male)	0.347	1.414	0.082	0.625	0.010
Type pf health facility (Hospital)	0.632	1.882	0.345	0.947	0.000
Monthly family income	0.349	1.418	-0.090-	0.828	0.123
Residency (Non -resident)	0.333-	1.395	0.009-	0.652	0.040
Availability of drinking water	0.780	2.181	0.067	1.591	0.018

^*^Significant at 0.05 significant level

## Discussion

This health facility-based study was conducted in two southern governorates in Yemen (Lahj and Abyan) where facing armed conflicts since 2015 affecting negatively upon the provision of the health services and exacerbate the existing malnutrition problem among children under five years. Out of the scope of the routine measurements of malnutrition in the community; this study focused on sick children seeking care in the health facilities for other health problems rather than malnutrition.

In this study; results revealed high prevalence of acute and chronic malnutrition (21.3% & 41.3% respectively) among studied sick children at 12–59 months of age in Lahj and Abyan governorates. This study focused on sick children observed in the outpatient clinics in both primary health care centers and hospitals. The reported rates of malnutrition in this study are higher than what, were reported in other developed or developing countries. Studies in developed countries reported significant proportion of malnutrition among hospitalized patients, Groleau V (2014) in their study in Canada reported that the prevalence of acute and chronic malnutrition among hospitalized children was 13.3% [19]. In another study in Canada in 2014; it was found that prevalence of acute malnutrition among children admitted to pediatric department was 6.9% while prevalence of chronic malnutrition was 13.4% [20]. Hulst J et al., (2004) concluded in their study in Netherlands that among critically ill patients it was found that the prevalence of malnutrition among children admitted to intensive care units (ICU) was 24% [21]. In developing countries, one study in Malaysia reported that the prevalence of acute and chronic under-nutrition among hospitalized children were 11% and 14% respectively [22]. In Pakistan the prevalence of stunting among children attending out-patient clinics was 21% [23]. In one controversial result from Tanzania; the prevalence of stunting and wasting was 8.37% & 1.41% respectively among children attending hospitals and primary care centers with predominance of boys' malnutrition over females [24]. This controversial results of low prevalence are due to methodological issue; investigators targeted all children attend to the health facilities either for seeking care or to attending to well child clinic for vaccination.

Severe acute malnutrition (SAM) has a significant contribution to child death if untreated, and may be exceeded the minimum Sphere standard (< 10%) especially in developing countries like Yemen and Ethiopia [25, 26].

In this study; the prevalence of SAM and MAM in sick children were 6.2% and 12.8% respectively, these figures are higher than the same indicators from community-based survey in Yemen (SAM 4.9%, MAM 8.4%) [27] and Ethiopia (SAM 3.6%, MAM 10.6%) prevalence of SAM in children under 5byears in Ethiopia [28].

Male children are more exposed to both acute and chronic malnutrition than females [29, 30]. In one study in Pakistan authors reported that significant higher prevalence of stunting in males than female children [23].

Concurrent wasting and stunting are an important problem among children under five years and it is considered risk factor to child mortality [31]. in this study; the prevalence of concurrent wasting and stunting was 6.85%, it is similar to prevalence in other developing countries like in Senegal it was 6.2% [31], 5.8% in Ethiopia [32] and 5% in Uganda [33].

Poverty is a critical determinant of malnutrition [34–36]. Yemen is a poor country, with poverty rates in Yemen increasing in recent years. For example, in 2018; the country ranked 178th out of 188 countries in the global Human Development Index ranking. Since 2015; Yemen was facing dramatic situation due to war and multi-epidemics and poverty [37]. Poverty can be both a cause and a consequence of malnutrition [38]. In this study; significant high prevalence chronic malnutrition among non-residents groups (IDPs, refugees and marginalized groups (so-called in Yemen Al-Mahmasheen).

The implication of this study is to give more attention for screening malnutrition among sick children as a routine examination. Studies reported that this routine screening is ignored in the routine medical care of sick children in many developing countries. In one study in Burundi (2019) it was found that only 3% of health workers screened children (6–59 months) for malnutrition [39].

There are certain limitations of this study. The study was limited to a selected health facilities in two governorates in southern Yemen due to logistics and accessibility issues. The study limited to patients attending health facilities in outpatient clinics, so critically ill-children were not included in the study.

## Conclusions

High acute and chronic malnutrition rates were identified among sick children seeking care in health facilities in lahj and Abyan governorates in Yemen. These higher malnutrion rates exceeded the SPHERE indicators of malnutrition. Boys are more exposed than girls to acute and chronic malnutrition. Gender (male) and type of health facility (Hospital) are predictors to acute malnutrition (wasting) while gender, type of health facility (Hospital) and residency (Non-resident) are predictors to chronic malnutrition (stunting). Authors advised that early detection of malnutrition in children at outpatient clinics should not be neglected. To avoid this ignorance to treat appropriately and to reduce mortality, authors recommended every sick child observed in outpatient or in-patents pediatric departments should be screening for malnutrition.

## Data Availability

All data sets are available and can be shared by requesting it from the corresponding author by email.

## Abbreviations

GAM: Global Acute Malnutrition
H/A: Height for Age
HAZ: Height for Age Z score
HUCOM: Hadhramout University College of Medicine
FAO: Food and Agriculture Organization
IDPs: Internally Displaced people
IPC: Integrated Phase Classification of Food Security
IRG: International Recognized Government
IRVD: The International War and Disaster Victims Protection Association
MAM: Moderate Acute Malnutrition
MUAC: Mid-upper arm circumference
OR: Odds Ratio
95%CI: 95% Confidence Interval
PHC: Primary Health Care
SAM: Severe Acute Malnutrition
SPSS: Statistical Package for Social Sciences
UNICEF: United Nations Children's Fund
WFP: World Food Program
W/H: Weight for Height
WHO: World health Organization
